# Japan's Telepsychiatry Dissemination: Current Status and Challenges

**DOI:** 10.2196/22849

**Published:** 2025-01-20

**Authors:** Shotaro Kinoshita, Taishiro Kishimoto

**Affiliations:** 1 Hills Joint Research Laboratory for Future Preventive Medicine and Wellness Keio University School of Medicine Tokyo Japan; 2 Graduate School of Interdisciplinary Information Studies The University of Tokyo Tokyo Japan

We read the recently published article titled “Issues in the Adoption of Online Medical Care: Cross-Sectional Questionnaire Survey” by Sugawara Y et al [1] with great interest. The authors conducted two surveys aimed at identifying the current status of telemedicine in Japan and the factors hindering its spread, from the perspectives of both patients and medical professionals providing it. Although the existence of bias cannot be denied because of the voluntary web-based survey, the findings were useful in understanding the current status of telemedicine in Japan.

As corrected in the original paper, the authors cited our study [2] and explained that telepsychiatry was reimbursed at the same or higher rate as in-person consultations in 15 of 17 regions worldwide. In this study, Japan was in the minority, with telepsychiatry reimbursement distinctly lower than face-to-face care. This remains an obstacle to the widespread use of telemedicine in Japan, not only in psychiatry but also across other medical specialties. Even before 2019, and despite deregulation following the COVID-19 pandemic, reimbursement rates for telemedicine have remained lower than those for face-to-face consultations [3].

In addition, telepsychiatry is more strictly regulated than telemedicine in other medical specialties. Even after the most recent deregulation in June 2024, there are still restrictions on prescription drugs and reimbursement rates for initial visits, as well as restrictions on the conditions under which physicians and medical facilities can obtain reimbursement.

The survey by Sugawara et al showed that patients and healthy individuals in psychiatry and psychosomatic medicine are interested in using telemedicine; however, the fact that medical institutions are either unable or unwilling to provide it, due to such strict regulations, is surely the biggest obstacle to its widespread use.

In addition, Sugawara et al emphasize the need to obtain the opinions of psychiatrists and psychosomatic medicine specialists regarding the feasibility of online medical care. In a 6-month randomized controlled trial comprising 199 patients with depression, anxiety, and obsessive-compulsive disorder at 19 medical institutions in Japan, we verified the noninferiority of the combined telepsychiatry group to the face-to-face treatment group [4]. The study also found no significant differences between the two groups on several secondary endpoints, including treatment retention and satisfaction. Furthermore, it confirmed that the telepsychiatry group required less time and incurred lower costs than the face-to-face treatment group. From these studies, we believe that the feasibility of telepsychiatry in the Japanese medical field has already been verified to a certain extent.

In Japan, while the demand for medical care is expected to increase due to further aging of the population, the dispersion and maldistribution of physicians and hospitals remains unresolved [5]. Since telemedicine is considered an effective measure to improve efficiency and equalize the provision of medical care, the government should ease regulations to make it easier for frontline medical professionals to use.
